# Influence of burnout on attitudes of ICU doctors and nurses towards liberalization of visiting polices

**DOI:** 10.1186/cc11097

**Published:** 2012-03-20

**Authors:** A Giannini, G Miccinesi, E Prandi, M Audisio, A Bencivinni, E Biagioni, E Castenetto, I Laganà, R Oggioni, V Porta, R Salcuni, A Sarti, MG Visconti, C Borreani

**Affiliations:** 1 Fondazione IRCCS Ca' Granda, Ospedale Maggiore Policlinico, Milan, Italy; 2 Istituto per lo Studio e la Prevenzione Oncologica, Florence, Italy; 3 Ospedale di Ciriè, Italy; 4 Ospedale Nuovo del Mugello, Borgo San Lorenzo, Italy; 5 Azienda Ospedaliera Universitaria di Modena, Italy; 6 Ospedale Civico, Chivasso, Italy; 7 Azienda Ospedaliera 'G. Salesi', Ancona, Italy; 8 Ospedale Civile, Legnano, Italy; 9 Ospedale di Ivrea, Italy; 10 Ospedale S. Maria Nuova, Florence, Italy; 11 Ospedale 'A. Uboldo', Cernusco sul Naviglio, Italy; 12 Istituto Nazionale per lo Studio e la Cura dei Tumori, Milan, Italy

## Introduction

The staff working in the ICU have a complex and stressful job and are at risk of burnout [1]. We conjectured that the presence of a burnout profile may also influence the views of ICU doctors and nurses regarding the liberalization of visiting policies. We investigated this issue in the course of a survey about the impact on ICU staff of liberalization of visiting policies.

## Methods

We administered an anonymous closed-question questionnaire to nurses and doctors at eight ICUs that were about to increase the daily visiting time to at least 8 hours, soliciting their views on policy changes in their unit. The ICU staff were asked to fill in the same questionnaire a year after implementation. On both occasions we also administered the Maslach Burnout Inventory (a 22-item self-completed questionnaire) to survey the incidence of burnout.

## Results

The first response rate was 91% (234/258), the second 76% (197/258). Most doctors and nurses gave a favourable opinion regarding changes to visiting policy in both the first (72%) and the second survey (71%). In both phases of the study, the percentage of respondents presenting a profile compatible with burnout was 36% and 41% respectively. In subjects without burnout there was a marked predominance of a favourable opinion (80% vs. 61%), and this favourable attitude was also maintained a year after the implementation of policy change (79% vs. 59%).

## Conclusion

The presence of burnout has a strong influence on the opinion of doctors and nurses regarding liberalization of visiting policies in the ICU. A favourable opinion predominates among ICU staff members without burnout symptoms. In preparing for and assisting the opening of ICUs it is important also to be aware of this aspect and to offer nurses and physicians appropriate support.
